# Radioligand therapy of metastatic castration-resistant prostate cancer: current approaches

**DOI:** 10.1186/s13014-018-1037-7

**Published:** 2018-05-23

**Authors:** Zool Hilmi Awang, Markus Essler, Hojjat Ahmadzadehfar

**Affiliations:** Department of Nuclear Medicine, University Hospital Bonn, Sigmund-Freud-Str. 25, 53127 Bonn, Germany

**Keywords:** PSMA, Prostate cancer, Radioligand therapy, Metastatic disease

## Abstract

Prostate Cancer is the forth most common type of cancer. Prostate-specific membrane antigen (PSMA) is anchored in the cell membrane of prostate epithelial cells. PSMA is highly expressed on prostate epithelial cells and strongly up-regulated in prostate cancer. Therefore it is an appropriate target for diagnostic and therapy of prostate cancer and its metastases. This article discusses several articles on radionuclide treatments in prostate cancer and the results on PSMA therapy with either beta or alpha emitters as a salvage therapy.

## Background

Prostate cancer is the fourth most common type of cancer affecting the European male population (excluding non-melanoma skin cancer) [1]. Currently, one in every six males are at risk of being affected by prostate cancer, and the risk of death because of metastatic prostate cancer is one in every 30 [2]. Castration-resistant prostate cancer (CRPC) is defined by disease progression despite castrated levels of testosterone, and may present as either a continuous rise in serum prostate-specific antigen (PSA) levels, the progression of pre-existing disease, and/or the appearance of new metastases [3].

In patients who fail the initial therapy with curative intent (i.e. radical prostatectomy, external beam radiotherapy [EBRT], brachytherapy) treatment options include androgen-deprivation therapy (ADT) along with chemotherapy in the case of disease progression [4]. A combination of ADT with docetaxel in hormone-sensitive patients improves median overall survival (OS) by 13.6 months compared to ADT alone [5, 6]. In CRPC patients, a more recent approach using abiraterone and enzalutamide prolongs median survival by up to 3.9 and 4.8 months, respectively [7, 8]. Chemotherapy treatment with docetaxel and cabazitaxel is often associated with side effects but prolongs OS for a few months [4, 9, 10]. Furthermore, treatment for diffused or painful bone metastases using radium-223-chloride (^223^Ra), which targets only the osteoblastic lesions and does not treat the nodal and visceral metastases, improves median OS by 3.6 months [11].

Positron emission tomography/computed tomography (PET/CT) using Gallium-68-labelled (Ga-68) ligands that target the prostate-specific membrane antigen (PSMA) is a sensitive and specific diagnostic method that is dedicated to poorly differentiated prostate cancer. Eiber et al. [12] reported that sensitivity increased to 100% with an increasing PSA velocity of 5 ng/ml/year or greater and with a Gleason score of eight or more. The use of PET/CT to target PSMA with Ga-68 for diagnostic and radioligand therapy (RLT) with Lutetium-177 offers a new theranostics approach using the same ligand for diagnostics and therapy [13]. Since 2013, an increasing number of centres worldwide has begun employing radioligand therapy (RLT) using ^177^Lu-PSMA [14–17].

The aim of this review is to discuss the current trend of using ^177^Lu-PSMA therapy, including dosimetry, side effects, treatment efficacy and survival rates, while referring to the literature and examining the prospects for prostate cancer therapy with targeted alpha therapy.

### Indications for RLT

**Fig. 1:**
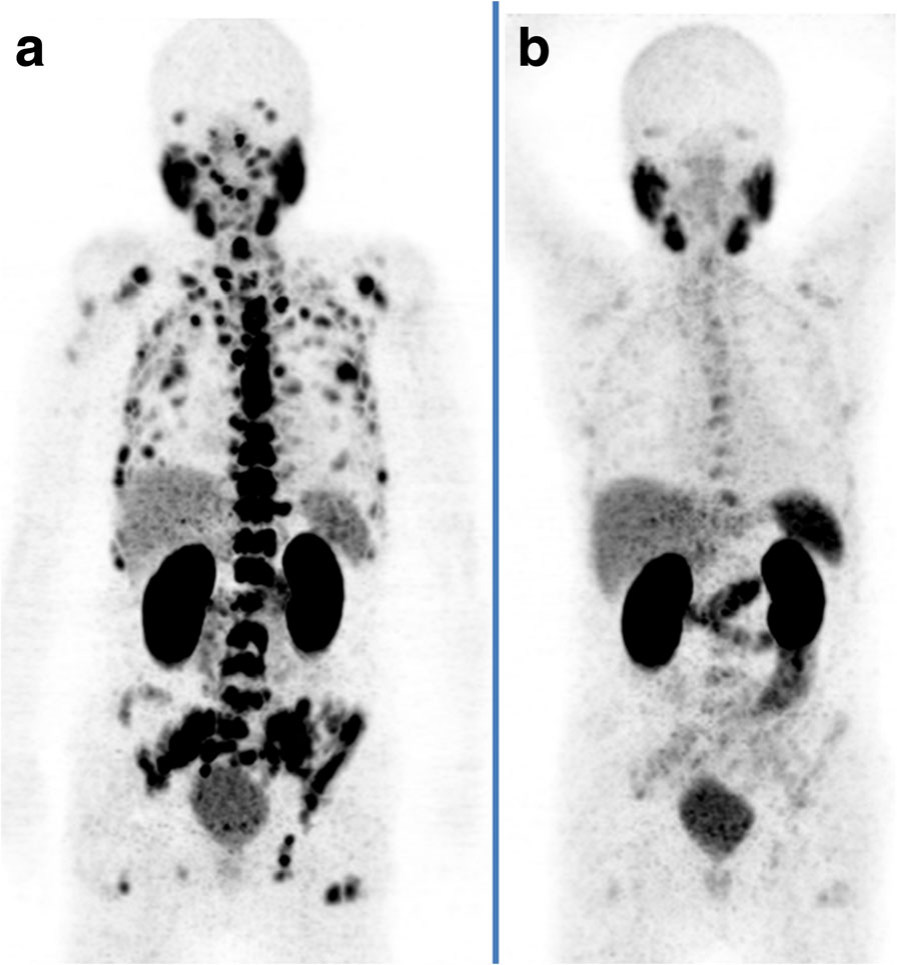
A 83-year-old patient with castration-resistant prostate cancer (Gleason-Score:9) and an increasing prostate-specific antigen (PSA) level. He had a history of prostatectomy and radiation therapy of prostate bed. The 68 Ga-PSMA PET scan showed a diffuse bone and bone marrow involvement (**a**). The PSA and ALP levels prior to the first cycle of Lu-PSMA therapy were 261 ng/ml and 659 U/l, respectively. The patient received 2 cycles of Lu-PSMA and the PSA level decreased continuously during cycles from 261 to 9.0 ng/ml (8 weeks after the second cycle). The ALP showed also a decreasing value from 659 to 81 U/l (8 weeks after the second cycle). The PSMA-PET (**b**) 8 weeks after the second cycle showed a significant response with significant regression of PSMA

Metastatic castration-resistant prostate cancer (mCRPC) patients can undergo treatment with taxane-based therapies (docetaxel and cabazitaxel) and with second line hormonal therapies (including enzalutamide and abiraterone). Both these therapies moderately improve patient survival time, but they are only temporarily effective and patients can develop resistance [18, 19]. Hence, more specific targeted therapies have to be developed for eliminating the prostate cancer visceral and bony lesions. Studies have shown that PSMA is overexpressed in around 90–100% of local prostate cancer lesions, along with many bony lesions and lymph node metastases. Furthermore, many studies have shown that the PSMA expression levels increase in the case of metastatic, high-grade and castration-resistant prostate cancer [20–22] (Fig. 1). The current essential inclusion criteria, as stated in the 2016 consensus recommendations of the German Society of Nuclear Medicine [23], cover:

1. histologically detected prostate carcinomas;
2. non-resectable metastases;
3. tumour progression under guidelines therapy;
4. detected PSMA expression of the tumour;
5. reasonable haematological function (leukocyte count > 2.0 × 10^9^/L, thrombocyte > 75 × 10^9^/L);
6. normal or slightly decreased renal function (creatinine < 2 x the upper standard limit);
7. sufficient liver function (aspartate aminotransferase [AST] or alanine aminotransferase [ALT] < 5 x the upper standard limit); and
8. a six-week interval with myelosuppressive therapy.

### Activity level

The standard administered activity of ^177^Lu-PSMA has varied across the literature as institutions undertake safety and toxicity trials [16]. Single injected doses have ranged from 3 to 9.3 GBq, with up to nine injections given to patients, generally at a minimum of six-week intervals [14, 24–31].

### Response rate

Up to 80% of patients with mCRPC will have a treatment response to ^177^Lu-PSMA shown by any PSA decline [14, 24–30, 32–34] (Table 1).

**Table 1:** Overview of published trials on treatment efficacy and OS

First author (n: number of patients)	Compound used	Activity	PSA fall ≥ 50%	CT (RECIST)	PSMA PET	Symptomatic response	Biochemical/radiological PFS	Overall survival
Vallabhajosula et al. 2005 [46]	^177^Lu-J591	0.3–2.7 GBq/m^2^for ^177^Lu	NA	NA	NA	NA	NA	NA
Lu (n: 35) = ^177^	^90^Y-J591	0.18–0.7 GBq/m^2^for ^90^Y						
n (28) = ^90^Y								
Zechmann et al. 2014 [37] (n: 28)	^131^I-MIP 1095	2.0–7.2 GBq	61%	NA	NA	23% CR	Median BPFS 126 days	NA
			PD 14%			61% PR		
Ahmadzadehfar et al. 2015 [14] (n: 10)	^177^Lu-PSMA-617	4.1–6.1 GBq	50%	NA	NA	NA	NA	NA
			PD 30%					
Ahmadzadehfar et al. 2016 [26] (n: 24)	^177^Lu-PSMA-617	4.1–7.1 GBq	42%	PR 40%	PR 80%	NA	NA	NA
			PD 21%	SD 55%	SD 0%			
				PD 5%	PD 20%			
Kratochwil et al. 2016 [32] (n: 30)	^177^Lu-PSMA-617	4–6 GBq	43–72%	NA	NA	NA	NA	NA
			PD 27%					
Baum et al. 2016 [51] (n: 56)	^177^Lu-PSMA I&T	3.6–8.7 GBq	59%	PR 20%	PR 56%	33% PR	Medial radiological PFS 13.7 months	
			PD 11%	SD 52%	SD 8%			
				PD 28%	PD 36%			
Rahbar et al. 2016 [25] (n: 74)	^177^Lu-PSMA-617	6 MBq	31%	NA	NA	NA	NA	NA
			PD 23%					
Rahbar et al. 2016 [39] (n: 28)	^177^Lu-PSMA-617	7 MBq	32–50%	NA	NA	NA	NA	29.4 vs. 19.7 weeks
			PD 20%					
Heck et al. 2016 [48] (n: 22)	^177^Lu-PSMA-I&T	7–7.8 GBq	33%	PR 11%	Integrated CR 5%	14% CR	Median PFS 175 days	NA
			PD 32%	SD 56%	SD 63%	42% PR		
				PD 33%	PD 32%			
Yadav et al. 2017 [52] (n: 31)	^177^Lu-PSMA-617	1.1–5.5 MBq	Mean pre and post		CR 33%	Analgesic score reduced from 2.5 to 1.8	Median PFS 12 months	Median OS 15 weeks
			275/41		PR 50%			
			PD 20%		SD 17% (*n* = 6)			
Fendler et al. 2016 [23] (n: 15)	^177^Lu-PSMA-617	6 MBq	60%	PR 27%	NA	NA	NA	NA
				SD 40%				
				PD 33%				
Kratochwil et al. 2016 [36] (n: 2)	^225^Ac-PSMA-617	4–6 GBq	100%	NA	NA	NA	NA	NA
Ahmadzadehfar et al. 2017 [27] (n: 52)	^177^Lu-PSMA-617	5.6–6 MBq	44% 1st cycle	NA	NA	NA	NA	Median OS 60 weeks
			59.6%					
			2nd cycle					
			59.6% 3rd cycle					
Rahbar et al. 2017 [16] (n: 145)	^177^Lu-PSMA-617	2–8 MBq	45%	PR 45%	NA	NA	NA	NA
				SD 28%				

*NA* not available, *MBq* mega becquerel, *CR* complete response, *PD* progressive disease, *PR* partial response, *OS* overall survival, *PFS* progressive free survival, *BPFS* biochemical progressive free survival, *PSMA* prostate specific membrane antigen, *PSA* prostate specific antigen, *RECIST* response evaluation criteria in solid tumors, *PET* positron emission tomography, *CT* computed tomography

Studies using ^177^Lu-PSMA-617 and ^177^Lu-PSMA-I&T have observed a reduction in PSA levels by 50% or more in 32–60% of patients. Moreover, 47% of patients have experienced a stable disease [14, 24–30, 32–34]. In 2016, a group from Heidelberg, Germany, started the first human treatments with ^255^Ac-PSMA-617 in two patients with red marrow infiltration and resistance to other therapies, and these patients showed complete responses to the therapy [35, 36]. A study by Zechmann et al., using a ^131^I-labelled PSMA ligand, showed a 50% or greater decline in PSA in more than 60% of patients [37]. This finding was in line with a recent study by Afshar-Oromieh et al. who studied 36 patients who had received PSMA-RLT with ^131^I-IMP-1095, and found the best therapeutic effect was achieved by the first therapy, which showed that PSA declined by more than 50% in 70.6% of the patients. The second and third therapies from their study showed reduced effectiveness [38].

### Predictors of the response

Ferdinandus et al. evaluated the prognostic value of various pre-therapeutic parameters on therapy response, based on changes in PSA after the first cycle of RLT. Their multivariate analysis of these parameters, which considered any decrease in PSA after 2 months, showed that patients with a high platelet count or a regular need for analgesics had a significantly worse response to the first RLT cycle. When a PSA decline of ≥ 50% was considered, patients with a regular need for analgesics showed a worse response in the multivariate analysis; however, other pre-therapeutic parameters had no impact on the response to RLT. In this study, the standard uptake value maximum of ^68^Ga-PSMA-11 was not a significant predictor of the response to RLT. One explanation for this could be that more aggressive tumours may express higher PSMA levels. However, despite the better uptake of ^177^Lu-PSMA-617 due to the rapid growth of metastases, the response rate did not correlate with the uptake, which could be due to different washout times of ^177^Lu-PSMA-617 in the respective metastases [29].

### Survival

Rahbar et al. reported a potential survival benefit of ^177^Lu-PSMA, where they matched the patient population (*n* = 28) to a historical cohort of 20 patients receiving the best supportive care (BSC) to examine potential survival benefits. Apart from the more heavily pre-treated patients and the more visceral metastases in the ^177^Lu-PSMA group, the groups were comparable. This finding highlights that the estimated median survival period was 29.4 weeks, being significantly longer than the survival time in the historical control group at 19.7 weeks [39].

In a study by Ahmadzadehfar et al. with 52 patients who underwent a total of 190 cycles of RLT, 80.8% of patients showed a decline in PSA levels 2 months after the first cycle, with 44.2% showing a PSA decline of ≥ 50%. The median OS was 60 weeks in all patients. The median OS was significantly longer for patients who showed a PSA decline after the first cycle compared to patients without a PSA decline (68 versus 33 weeks respectively) [27]. In another study from the same group, 100 patients who received a total of 347 cycles of ^177^Lu-PSMA (median three cycles) were analysed. All patients had a history of therapy with abiraterone or enzalutamide, or both. In total, 70% of the patients had at least one line of chemotherapy and 36% had a history of radionuclide therapy with ^223^Ra. Sixty-nine patients showed a decline in PSA 2 months after the first cycle, and 38 of these patients showed a PSA decline of ≥ 50%. The median OS was 60 weeks. In the multivariate analysis, the median OS was significantly longer in those patients without hepatic involvement, with high levels of albumin and haemoglobin (Hb), and with low levels of AST. Moreover, in the univariate analysis, a PSA decline after the first RLT, as well as any decline > 50%, were significant predictors of a longer OS. A decline in PSA levels of more than 14% was the most important response parameter with regard to OS [40]. In a bicentric study, 104 patients were treated with 351 cycles of ^177^Lu-PSMA-617. All of them had a history of therapy with at least one line of chemotherapy as well as either abiraterone or enzalutamide. Thus, in this study, the patients received all recommended guideline therapies. A PSA decline occurred in 70 (67%) patients, with a PSA decline ≥ 50% in 34 (33%) patients after the first cycle. The median OS was 56.0 weeks (95% CI: 50.5–61.5). Any initial PSA decline, an initial alkaline phosphatase (ALP) < 220 U/L and a cumulative injected activity of ≥18.8 GBq were associated with a longer survival. A step-by-step analysis revealed a PSA decline of ≥ 20.9% as the most noticeable cut-off prognosticating longer survival, which remained an independent prognosticator of improved OS in the multivariate analysis [41]. These studies have shown that responders to PSMA therapies live longer than non-responders, and a PSA response should not necessarily be defined as a PSA decline of > 50%. Interestingly, prior therapies, such as chemotherapy, had no impact on OS.

### Dosimetry

The distribution of small molecules of PSMA ligands in tissue is quick and, over time, the uptake in prostate cancer tissue increases, whereas the uptake in healthy tissue declines [42]. In normal healthy tissue, salivary glands have the highest PSMA binding, followed by normal kidney tissue.

Kabasakal et al. [15] reported their dosimetry results with ^177^Lu-PSMA-617 and showed the highest radiation estimated doses in parotid glands and kidneys. Calculated radiation-absorbed doses per megabecquerel were 1.17 ± 0.31 mGy for parotid glands and 0.88 ± 0.40 mGy for kidneys. The radiation dose given to bone marrow was significantly lower than those of kidney and parotid glands (*p* < 0.05). The calculated radiation dose to bone marrow was 0.03 ± 0.01 mGy/MBq.

These results were reproduced by Delker et al. [43] who reported their dosimetry results with ^177^Lu-PSMA-617 and calculated a mean absorbed dose to bone marrow, kidneys, liver, spleen, and salivary glands of 0.012 Gy/GBq, 0.6 Gy/GBq, 0.1 Gy/GBq, 0.1 Gy/GBq and 1.4 Gy/GBq respectively.

There are several profound fears with regards to the damage caused to salivary glands. Based on external beam radiotherapy (EBRT) data, irreversible damage to salivary glands occurs after the administration of 30–40 Gy. With a mean absorbed dose of 1.4 Gy/GBq of ^177^Lu-PSMA-617, and the absence of permanent xerostomia or hypogeusia in the initial treatment studies, the salivary glands do not appear to be a dose-limiting organ [43].

The second fear concerns the absorbed dose to kidney tissues [15, 43], where, based on the EBRT data, a dose of 23 Gy may result in permanent damage. The mean absorbed kidney dose of ^177^Lu-PSMA is 0.53–0.8 Gy/GBq, quite like the absorbed kidney dose mentioned in the published data on ^177^Lu-DOTATATE (0.64 ± 0.16 Gy/GBq) [44].

In a study with 135 patients undergoing diagnostics using ^68^Ga-PSMA PET-CT, Gaertner et al. [45] compared three groups of patients with low, moderate, and high tumour loads according to their tumour volume. Their results indicate that patients with high tumour loads may receive less toxicity in their non-target organs.

As a result of previous findings, the safety and efficacy of targeted radionuclide therapies can be improved using patient-specific dosimetry, which may help to guide successful tumour dosing and act as an early indicator of organ toxicity.

### Toxicity

#### Myelosuppression

Myelotoxicity is a classic non-stochastic (deterministic) effect. This effect is characterised by a sigmoidal, dose-response relationship [46].

A report from a German multicentre study [16] showed that grade 3–4 hematologic adverse events occurred in 18 of 145 patients (12%). Furthermore, one (0.7%) patient experienced severe leukopenia, 11 (8%) patients experienced anaemia, two (2%) patients experienced thrombocytopenia, and four (3%) patients had a combination of these conditions.

Ahmadzadehfar et al. [28] showed in a retrospective analysis of 49 patients, who had undergone three cycles of RLT with at least 2 months of follow up after the last cycle, that there was no CTC 4° haematotoxicity in the entire study population. Relevant anaemia, thrombocytopenia and leukopenia (CTC 3°) occurred during the observation period after the third cycle in four (8.2%), three (6.1%) and zero patients, respectively. The patients were divided into two groups with regard to their history of therapy with ^223^Ra. Group 1 included 20 patients who had received therapy with ^223^Ra (median six cycles) prior to ^177^Lu-PSMA-617 therapy. Group 2, which was the control group regarding haematotoxicity, was comprised of 29 patients without any history of a bone-targeted radionuclide therapy. There was no significant difference between the groups regarding relevant haematotoxicity. Thus, according to the results of this study, performing repeated cycles of ^177^Lu-PSMA-617 after ^223^Ra seems to be safe, with a very small probability of haematotoxicity [28].

#### Renal toxicity

Due to the physiological expression of PSMA in kidneys, many researchers have been concerned about probable radiation toxicity to the kidneys. Along with glomerular filtration rate (GFR) and creatinine levels, renal scintigraphy should be performed with Tc-MAG3 before therapy for overruling any significant obstructive disease. Any relevant obstructive disease must be treated in order to decrease the radiation dose to the diseased kidneys. Recently, Yordanova et al. [47] reported on 55 patients treated with ^177^Lu-PSMA-617, where 14 (25%) showed a (sub-)acute toxicity of CTC 1° and only one patient had a CTC 2° according to the creatinine value. No grade 3–4 acute loss of renal function was detected, and this was in line with the German multicentre study [16]. A decreased GFR was observed in 16 patients (29%) where four had CTC 1° and 12 had CTC 2° toxicity [47]. It has been suggested that conditions that may affect renal function and increase the radiation absorbed dose to the kidneys occur in patients who are older-age men, who have had prior chemotherapy and who have accompanying diseases, such as hypertension [47] (Table 2).

**Table 2:** Published data on myelosuppression and complaints after therapy

	Anemia				Thromcytopenia				Leucopenia			
First Author year	Grade 1	Grade 2	Grade 3	Grade 4	Grade 1	Grade 2	Grade 3	Grade 4	Grade 1	Grade 2	Grade 3	Grade 4
Deb et al. 1996	X	X	-	-	X	X	X	-	X	X	X	-
Vallabhajousula et al. 2005	-	-	-	-	X	X	X	X	-	-	-	-
Bander et al. 2005	-	-	-	-	X	X	X	X	-	-	-	-
Tagawa et al. 2013	X	X	X	-	X	X	X	X	X	X	X	X
Zechmann et al. 2014	-	-	-	-	X	X	X	-	X	X	X	_
Ahmadzadehfar et al. 2016	X	X	X	-	X	-	-	-	X	X	-	-
Kratochwil et al. 2016	X	X	X	-	X	X	X	-	X	X	-	-
Baum et al. 2016	X	X	-	-	-	-	-	-	X	X	-	-
Rahbar et al. 2016	X	X	-	-	X	-	-		X	-	-	-
Rahbar et al. 2016	-	-	X	-	X	-	-	-	-	-	-	-
Heck et al. 2016	X	X		-	X	X	-	-	X	X	-	-
Yadav et al. 2016	X	X	X	-	-	-	-	-	-	-	-	-
Fendler et al. 2016	X	X	-	-	X	X	-	-	X	X	X	-
Kratochwil et al. 2016	X	X	-	-	X	-	-	-	X	-	-	-
Ahmadzadehfar et al. 2017	X	X	X	-	X	X	X	-	X	X	-	-

#### Salivary glands

Although the salivary glands contain highly differentiated cells and their proliferation rate is slow, they are exceptionally radiosensitive organs. Due to the high binding of PSMA ligands, damage to the salivary glands and the development of xerostomia is a frequent side effect of radiation therapy which decreases the patient’s quality of life. In the studies by Ahmadzadehfar et al. [26–28, 40], Heck et al. [48], and Rahbar et al. [14, 25, 41], patients received an ice pack collar for 30 min before and for up to 4 h after the administration of ^177^Lu-PSMA-617 to induce vasoconstriction and reduce PSMA binding to the salivary glands. Transient xerostomia or hypogeusia occurred in 4–37% of patients with or without an ice pack collar [16, 26, 48].

#### Toxicity of ^225^Ac-PSMA

As stated earlier, ^225^Ac-PSMA therapy can be used in the patients who are unresponsive to ^177^Lu-PSMA therapy or show pronounced bone marrow infiltration. ^225^Ac-PSMA radiation consists of short-ranged alpha particles which kill tumour cells but spare the bone marrow cells. In a recent study, many patients reported the success of their ^225^Ac-PSMA therapy [36]. Also, in the case of the preclinical models of neuroendocrine tumours, ^225^Ac-DOTATOC was very effective in controlling the tumours, thus indicating that ^225^Ac-PSMA therapy was very effective [49]. In one recent study, researchers treated 40 patients using ^225^Ac-PSMA therapy and noted that four patients had to discontinue their treatment because of xerostomia. Twenty four patients (63%) showed more than a 50% decrease in their PSA levels while 33 (87%) patients showed some PSA response. The median duration for tumour control after the last-line of ^225^Ac-PSMA-617 therapy was 9 months, while five patients endured a response for ≥ 2 years [36, 50]. None of the patients showed any hematologic toxicity, and xerostomia was the only clinical side effect observed [36].

## Conclusions

^177^Lu- and ^225^Ac-based PSMA-targeted therapies appear to be promising and effective treatments for advanced prostate cancer. The new types of diagnostic tracers show a high sensitivity and specificity in the imaging of prostate cancers, even in patients with very low PSA levels, which has helped in the diagnostics, especially for staging and follow up during RLT. Therefore, prospective randomised trials are required to determine the impact of ^177^Lu-PSMA on survival, toxicities, dosimetry, and to rigorously assess the clinical benefits compared to other treatments for prostate cancer, including chemotherapy, EBRT, and androgen blockade. We suggest that a new type of nuclear medicine specialist should be established to perform and further develop radionuclide therapy: theranostics specialists should be trained in the fields of radionuclide treatment, radionuclide imaging, oncology, and radiation therapy to cover all aspects of these complex therapies and to ensure that this new treatment option is accepted worldwide.

## Abbreviations

ALT: Alanine aminotransferase
AST: Aspartate aminotransferase
CRPC: Castration-resistant prostate cancer
CTC: Common toxicity criteria
EBRT: External beam radiotherapy
Hb: Haemoglobin
MBq: Megabecquerel
OS: Overall survival
PET/CT: Positron emission tomography/computed tomography
PSA: Prostate-specific antigen
PSMA: Prostate-specific membrane antigen
RLT: Radioligand therapy
SUV: Standard uptake value
